# Liquid Chromatography–Electrospray Ionization Tandem Mass Spectrometry Estimation of Quercetin-Loaded Nanoemulsion in Rabbit Plasma: *In Vivo*–*In Silico* Pharmacokinetic Analysis Using GastroPlus

**DOI:** 10.1021/acsomega.3c00429

**Published:** 2023-03-20

**Authors:** Sabya Sachi Das, Priya Ranjan Prasad Verma, Viswanathan Sekarbabu, Satyajit Mohanty, Ashok Kumar Pattnaik, Janne Ruokolainen, Kavindra Kumar Kesari, Sandeep Kumar Singh

**Affiliations:** † Department of Pharmaceutical Sciences and Technology, Birla Institute of Technology, Mesra, Ranchi 835215, Jharkhand, India; ‡ School of Pharmaceutical and Population Health Informatics, DIT University, Dehradun 248009, Uttarakhand, India; § Innospecs Bioresearch Private Limited, Rajakilpakkam, Chennai 600073, Tamil Nadu, India; ∥ Department of Applied Physics, School of Science, Aalto University, 00076 Espoo, Finland; ⊥ Faculty of Biological and Environmental Sciences, University of Helsinki, Biocentre 3, Helsinki 00014, Finland

## Abstract

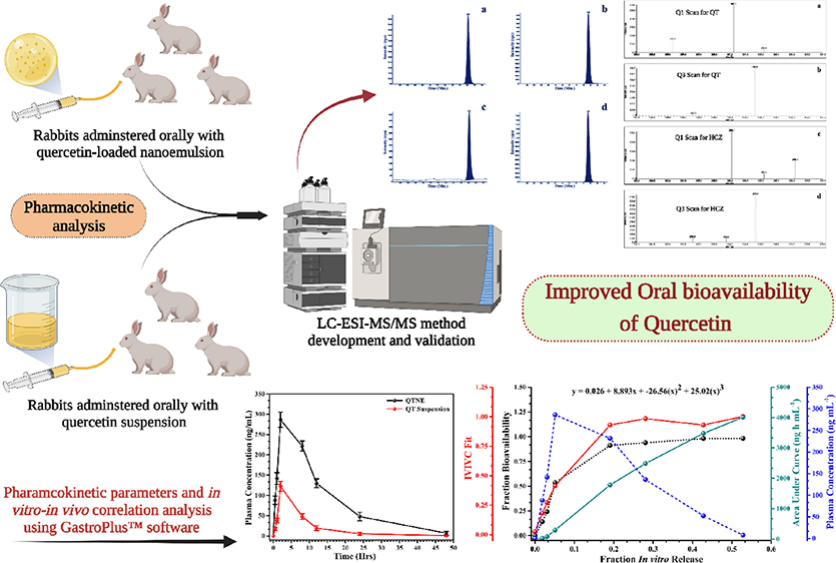

In the present study, we developed and validated a rapid, specific, sensitive, and reproducible liquid chromatography–electrospray ionization tandem mass spectrometry method for quantifying quercetin (QT) in rabbit plasma using hydrochlorothiazide as the internal standard. Animals were orally administered with optimized QT-loaded nanoemulsion (QTNE) and QT suspension (QTS), equivalent to 30 mg/kg, to the test and control group, respectively. The blood samples were collected at pre-determined time points up to 48 h. The linearity range was from 5 to 5000 ng mL^–1^ with *R*^2^ = 0.995. Further, we analyzed the various pharmacokinetic parameters and established the *in vitro*–*in vivo* correlation (IVIVC) of QTNE using GastroPlus software. The method was successfully developed and validated, and when applied for the determination of QT in rabbit plasma, it exhibited an increase in *C*_max_ from 122.56 ng mL^–1^ (QTS) to 286.51 ng mL^–1^ (QTNE) (2.34-fold) and AUC_0–48_ from 976 ng h mL^–1^ (QTS) to 4249 ng h mL^–1^ (QTNE) (4.35-fold), indicating improved oral bioavailability QT when administered as QTNE. Statistical analysis revealed that the Loo–Riegelman method (two-compartmental method) best fitted the deconvolution approach (*R*^2^ = 0.998, SEP = 4.537, MAE = 2.759, and AIC = 42.38) for establishing the IVIVC. In conclusion, the established bioanalytical method and IVIVC studies revealed that QTNE is a potential carrier for the effective delivery of QT with enhanced oral bioavailability.

## 1 Introduction

Quercetin (QT, 3,3,4,5,7-pentahydroxyflavone) is a polyphenolic flavonoid that is abundantly found in various plant sources, including vegetables (e.g., onion and broccoli), fruits (e.g., apple and blueberry), and also in herbs.^1,2^ QT exhibits potential antioxidant properties, leading to its diverse biological actions, including anticancer, antimicrobial, antiproliferative, anti-inflammatory, and neuroprotective effects and others.^3^ Although QT exhibits various pharmacological actions, its applications are restricted due to its low mucosal permeability leading to low oral bioavailability (<17% in rats and ∼1% in humans).^4^ The low bioavailability of QT could be due to its lipophilic behavior and its high affinity for both the Cyp450 (CYP3A) and the intestinal efflux pumps (e.g., P-gp and MRP2), abundantly found in the GIT epithelium.^5^ Therefore, to improve the oral bioavailability of QT, it becomes essential to incorporate it within a stable nanocarrier-based delivery system with negligible or no systemic toxicity.

Several nanotechnology-mediated strategies such as nanoemulsions, liposomes, polymeric nanoparticles, and micelles for QT have been established to improve the solubility, permeability, and bioavailability.^6^ Lipid-based nanotechnological strategies have shown potential effects in improving the therapeutic effects of encapsulated polyphenolic compounds, including QT.^6^ Nanoemulsions (NEs), a type of lipid-based nanocarrier, are formed from the dispersion of two immiscible liquid phases (oil and water) that forms oil-in-water (o/w) or water-in-oil (w/o nanodroplets) systems stabilized with the amphiphilic surfactant or cosurfactant.^4^ The compatibility between oil and surfactant and the encapsulated drug moiety plays a crucial role in maintaining the stability of the NE system.^7^ The estimation of possible interactions (either negative or positive) between the drug and excipients exhibits a vital role during preformulation studies for the development of stable NEs. Moreover, the interplay between drugs and excipients significantly improves the drug’s pharmacokinetic parameters by improving the pharmaceuticals.^8^

Pharmacokinetic characteristics of drug molecules play a crucial role in understanding their *in vivo* performance and mechanism of action. Usually, the flavonoids are consumed orally and may be bio-transformed *via* intestinal microbiota and metabolized within the liver.^9−11^ During this process, metabolites (in plasma) may produce similar, more potent, or weaker effects than those with the parent drug moiety.^12,13^ Various analytical approaches have been developed and implemented to understand the drug release mechanism of QT in biological samples using several analytical methods, including high-performance liquid chromatography (HPLC) with UV detection,^14,15^ fluorescence detection,^16^ electrochemical detection,^17^ and liquid chromatography–electrospray ionization tandem mass spectrometry (LC-MS/MS) analysis.^18−20^ In another study, the researchers compared the pharmacokinetic parameters of QT with QT-3-*O*-β-glucuronide after oral administration (100 mg/kg dose) in rats using the UHPLC-MS/MS method.^21^ In a recent study, the QT-chitosan oligosaccharide-based amorphous dispersions exhibited improved oral bioavailability (1.64–2.25 times) compared to pure QT-treated male Sprague Dawley rats, evaluated by the HPLC method.^14^

Herein, we developed and validated a rapid, specific, sensitive, and reproducible liquid chromatography–electrospray ionization tandem mass spectrometry (LC-ESI-MS/MS) method for quantifying QT in rabbit plasma after oral administration of quercetin-loaded nanoemulsion and quercetin suspension. A comparative analysis of pharmacokinetic parameters of QT from nanoemulsion and suspension and the IVIVC studies for QTNE was performed using the GastroPlus (Simulations Plus Inc., Lancaster, CA, USA) software.

## 2 Materials and Methods

### 2.1 Materials and Chemicals

Quercetin (QT) and methanol (HPLC grade) were purchased from Sigma-Aldrich Chemicals, USA. Hydrochlorothiazide (HCZ) was procured from Clarsynth Labs, Mumbai, India. Capmul MCM NF (CAP MCM NF) and Cremophor RH40 (CR RH40) were obtained from Gattefosse (Saint-Priest, Cedex, France) and BASF (Ludwigshafen, Germany), respectively. Acetonitrile (CH_3_CN) and ammonium formate (NH_4_HCO_2_) were procured from Thermo Fisher Scientific, India. HPLC grade water was obtained from Rankem, India. All other chemicals and reagents were of analytical grade.

### 2.2 Preparation of Quercetin-Loaded Nanoemulsion

The optimized quercetin-loaded nanoemulsion (QTNE) was prepared as mentioned in our earlier published work.^4^ The QTNE comprises QT (drug, 10 mg), CAP MCM NF (oil, 250 mg), and CR RH 40 (surfactant, 250 mg). The QTNE formulation was prepared by mixing the aqueous phase (surfactant + Milli Q water, 10 mL) into the oil phase (drug + oil) followed by stirring (IKA C-MAG HS 7, Germany) at 500 rpm. Further, the system was homogenized (IKA T25, ULTRA TURRAX, Germany) at 11,000 rpm for 20 min.

### 2.3 LC-ESI-MS/MS Method Development

#### 2.3.1 Chromatographic and Tandem Mass Conditions

**Figure 1:**
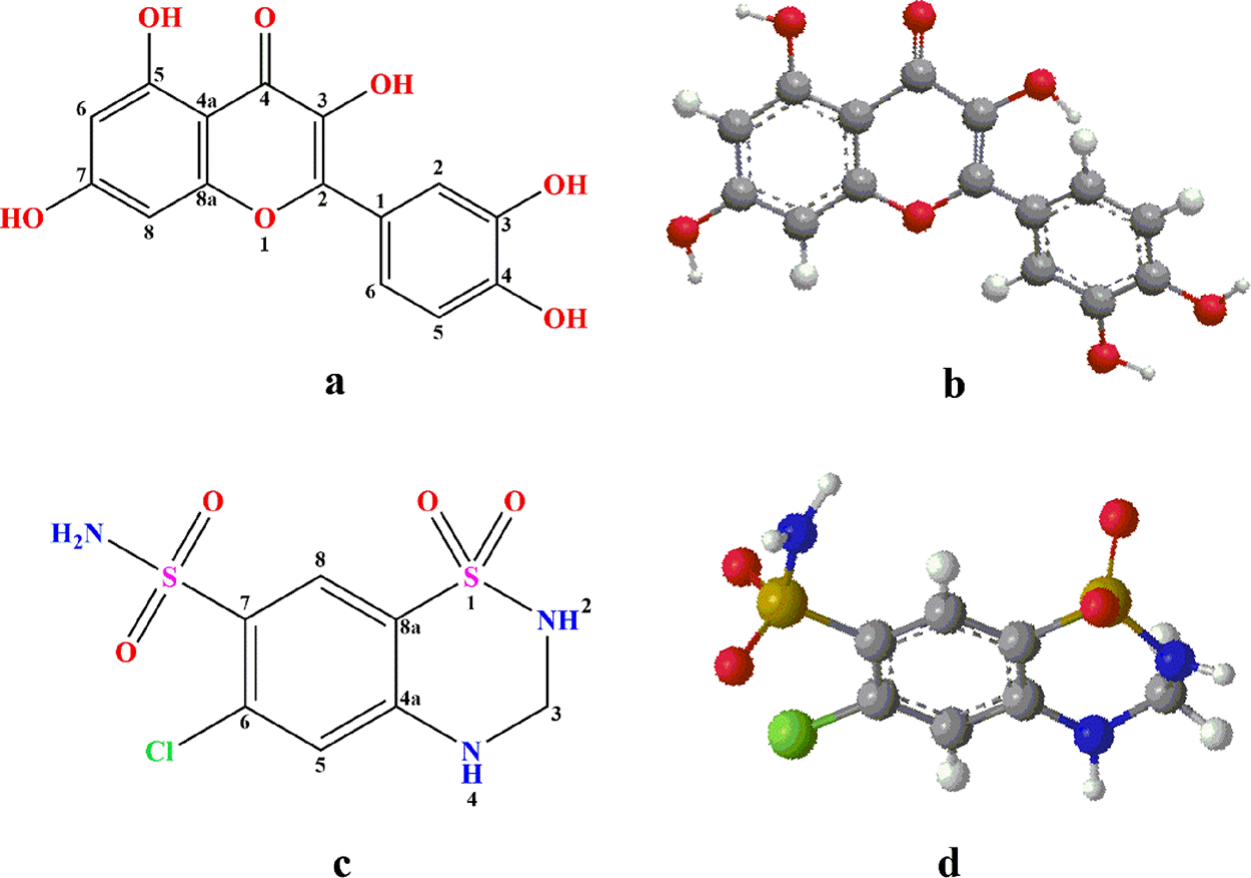
Chemical structure of quercetin: two-dimensional (a) and three-dimensional (b); and hydrochlorothiazide: two-dimensional (c) and three-dimensional (d).

The samples were analyzed using an LC-MS/MS system, ultra-fast liquid chromatography from Shimadzu (HPLC 20 AD LC system, Japan), and Triple quad API 4000 MS/MS from AB Sciex (Applied Biosystem, Foster City, Canada). The analytical column used for separation was a Hypersil Gold column (150 mm × 4.6 mm, 5 um, Thermo Fisher Scientific, Waltham, MA, USA), and the column oven temperature was set at 40 °C. During analysis, acetonitrile with 0.1% formic acid (80:20, v/v) was employed as the mobile phase. The flow rate of the mobile phase was set as 0.500 mL/min, and a 10.0 μL sample volume was used for analysis. Turbo ion spray ionization (ESI) in negative mode was used as an ionization technique. The optimal interface circumstances were (a) an ion spray voltage of −4500 V, (b) interface temperature of 400 °C, (c) delustering potential of −50 V, (d) entrance potential of −10 V, (e) collision energy at −30 V, and (f) collision exit potential at −15 V. The selected reaction monitoring (SRM) ion transitions were Q1:Q3-301.20/150.80 *m*/*z* for QT (Figure 1a,b) and Q1:Q3-296.10/204.80 *m*/*z* for HCZ (internal standard; Figure 1c,d), with a scan time of 200 ms per transition.

#### 2.3.2 Preparation and Calibration of Standard Stock Solutions

The stock solutions (100 μg mL^–1^) of QT and HCZ (internal standard) were prepared by dissolving 2.0 mg of each in methanol (20.0 mL). The standard calibration curves of QT in plasma samples were plotted in the 5–5000 ng mL^–1^ concentration range. Further, the quality control (QC) samples were prepared by diluting QT in the plasma and were represented as 11.356 ng mL^–1^ (low concentration; LQC), 1880.966 ng mL^–1^ (medium concentration; MQC), and 3761.932 ng mL^–1^ (high concentration; HQC).

### 2.4 Method Validation

A total of seven calibration standards in a concentration range of 5–5000 ng mL^–1^ were selected for evaluating the linearity. The calibration curves were plotted for the *y* axis (peak ratio of QT and HCZ) vs the *x* axis (concentration of QT) using a weighted factor (1/*x*). The LLOQ represents the lowest concentration over the calibration curve and should have an accuracy (within ±20%) and precision (<20%).

For three independent days, accuracy and precision were investigated for the drug’s LQC, MQC, and HQC concentrations. The inter- and intra-day precision were measured by percent relative standard deviation (%RSD). The accuracy was determined using eq 1:^19^
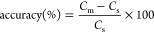
1where *C*_m_ is the mean observed concentration and *C*_s_ is the spiked concentration.

The extraction recovery of QT was estimated by correlating the QT samples’ (LQC, MQC, and HQC) responses with the analyte’s responses from the post-extracted plasma samples at equivalent concentrations. The recovery studies for QT were achieved at three concentration levels (11.2560, 1880.9660, and 3761.9320 ng mL^–1^) in the rabbit plasma. The matrix effects were studied at LQC, MQC, and HQC by relating the peak area of the treated plasma samples and spiked later to acquire three QC level concentrations (*A*_P_) with the peak area of the standard solutions (*A*_S_). Equation 2 was used to determine the matrix effect.
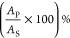
2

QT’s freeze–thaw stability in the rabbit plasma was studied for three QC level concentrations (LQC, MQC, and HQC) after three freeze (−20 °C)–thaw cycles. Analysis and comparison of the freeze-thawed samples and freshly prepared QC samples were made by monitoring the peak areas of the respective samples.

### 2.5 Pharmacokinetic Studies

**Figure 2:**
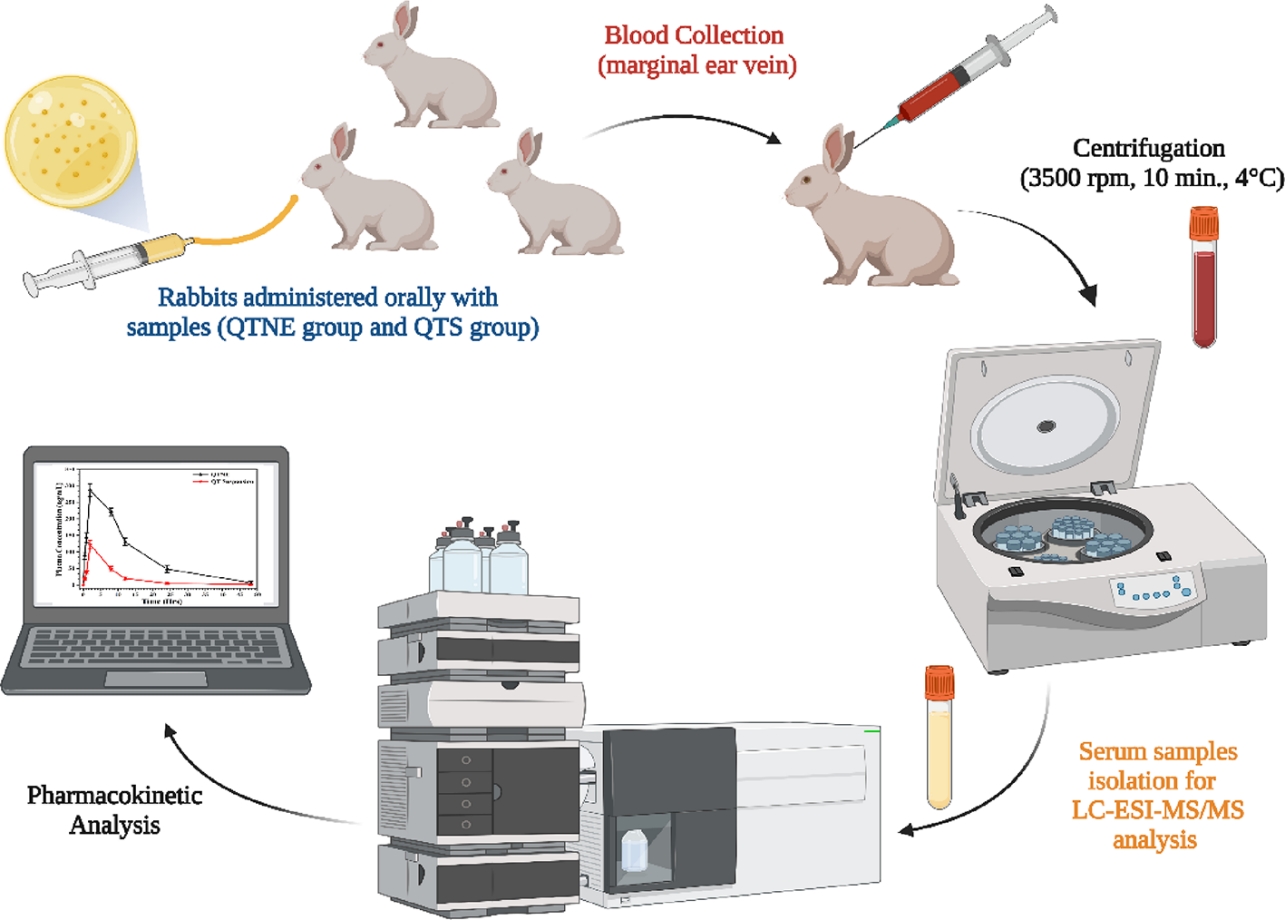
Illustration describing the preparation steps involved in sampling of rabbit plasma.

To perform the pharmacokinetic studies of QTNE and QTS, adult male New Zealand white rabbits (1.3 ± 0.8 kg body weight) were used. The animals were acclimatized for 7 days under standard conditions (25 ± 2 °C, 60% RH) before conducting the study, and the necessary diet and water were provided *ad libitum*. The rabbits were fasted overnight (before experimentation) and were arbitrarily divided into two groups (*n* = 3): QTNE-treated animals (group I) and QTS-treated animals (group II). QTS was prepared by mixing an adequate amount of QT in an aqueous suspension comprising 0.5% w/v Na-CMC. The samples were orally administered to all animals with a 30 mg/kg QT dose. The protocols for the study were followed as per the CPCSEA (Committee for Control and Supervision of Experiments on Animals) guidelines after approval from the Institutional Animal Ethics Committee, Department of Pharmaceutical Sciences and Technology, BIT, Mesra, Ranchi (1972/PH/BIT/42/18/IAEC*). The blood samples were withdrawn from the marginal ear veins from treated animals at time points of 0.5, 1, 2, 8, 12, 24, and 48 h, collected in heparinized tubes, and centrifuged at 3500 rpm for 10 min, and the corresponding plasma samples were stored at −20 °C. The samples were later analyzed using the LC-ESI-MS/MS technique, and the process is illustrated in Figure 2.

Further, the pharmacokinetic parameters including *C*_max_, *t*_max_, AUC_0–*t*_, AUC_0–∞_, AUMC, *t*_1/2_, MRT, *K*_a_, *K*_e_, and micro-constants were measured using the PKPlus module of GastroPlus software. Furthermore, different pharmacokinetic compartment models, including 1C, 2C, and 3C, were statistically analyzed, and the best-fit model was selected considering high *R*^2^ and low SC and AIC values.^22^

### 2.6 *In Vitro*–*In Vivo* Correlation Determination

The foremost goal behind the *in vitro*–*in vivo* correlation (IVIVC) study is to act as a substitute for *in vivo* studies. IVIVC studies impart potential benefits for the pharmaceutical industries as it saves time, improves cost efficiency, and assists in formulation optimization. IVIVC studies were established through several steps, including (i) generation of *in vitro* cumulative drug release data (*in vitro*); (ii) development of PDCT profiles (*in vivo* dosing); and (iii) generation of an *in vivo* dissolution data through numerical deconvolution of the PDCT profiles. Finally, the dissolution profile (*in vivo*) was compared with the experimental dissolution data (*in vitro*).^23^

The IVIVC module of GastroPlus software was used to establish the correlation between *in vitro* dissolution study data, as reported in our previously published work,^4^ and *in vivo* plasma concentration data for QTNE and QTS. The PDCT profile was studied by 1C, 2C, and 3C methods using the PKPlus module of GastroPlus software, and the relevant pharmacokinetic parameters were analyzed. Further, deconvolution methods were employed for establishing correlations, and based on the values of *R*^2^, SEP, and MAE, the best-fit correlation model was selected for establishing IVIVC. Additionally, convolution was performed and was assessed based on the statistical findings of MAPPE for two prime pharmacokinetic parameters, i.e., *C*_max_ and AUC.

## 3 Results and Discussion

### 3.1 Method Development and Validation

**Figure 3:**
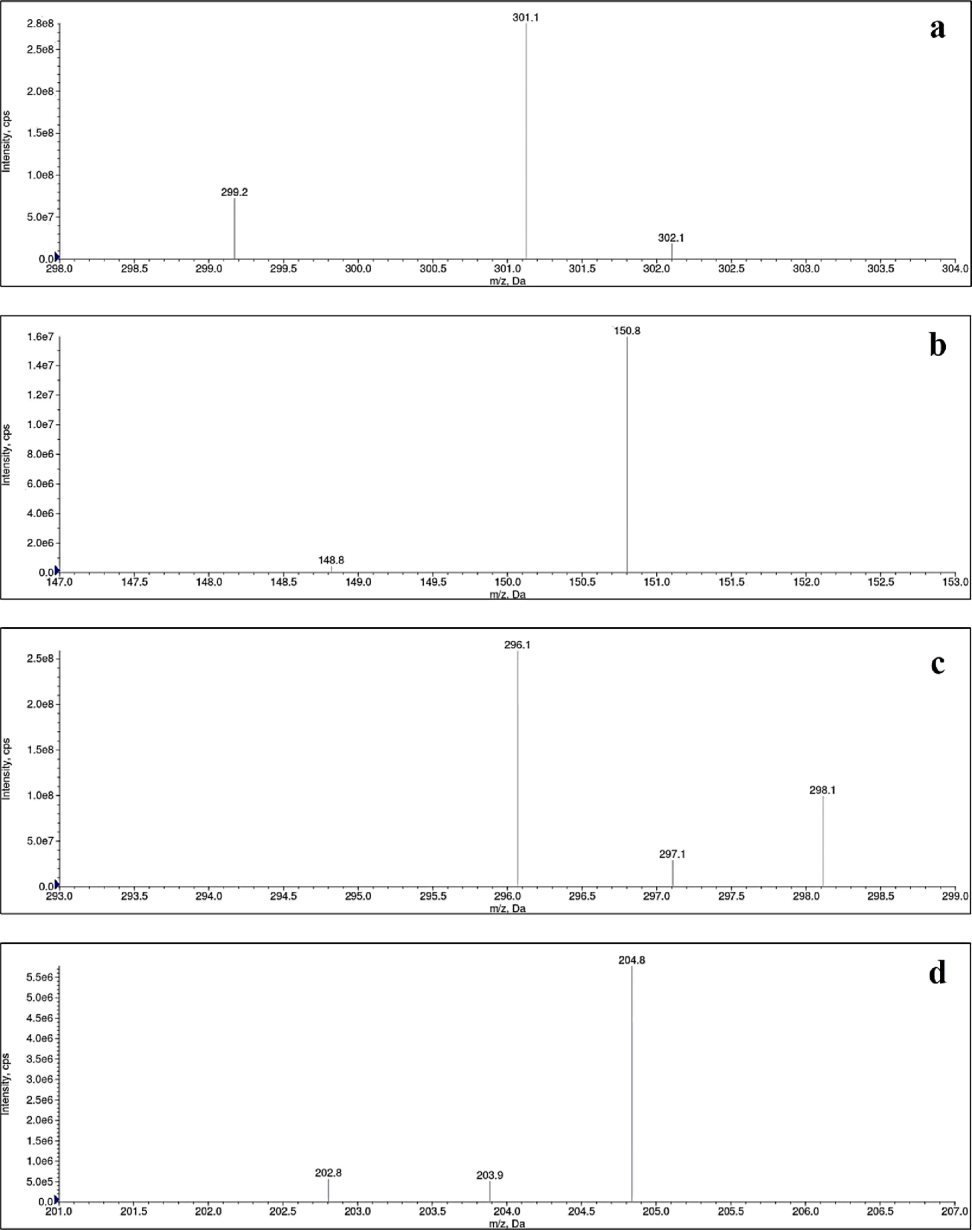
Characteristic mass spectra of quercetin: deprotonated molecule (a) and product ion (b); and hydrochlorothiazide: deprotonated molecule (c) and product ion (d).

The negative ionization mode of LC-ESI-MS/MS was used for analyte quantification to achieve higher sensitivity and selectivity of data. The SRM pair detected the ion transitions Q1:Q3-301.20/150.80 *m*/*z* for QT (Figure 3a,b) and Q1:Q3-296.10/204.80 *m*/*z* for HCZ (Figure 3c,d), with a scan time of 0.20 s per transition. The retention times for QT and HCZ were found at 2.02 and 1.94 min, respectively (total run time: 2.50 min).

**Figure 4:**
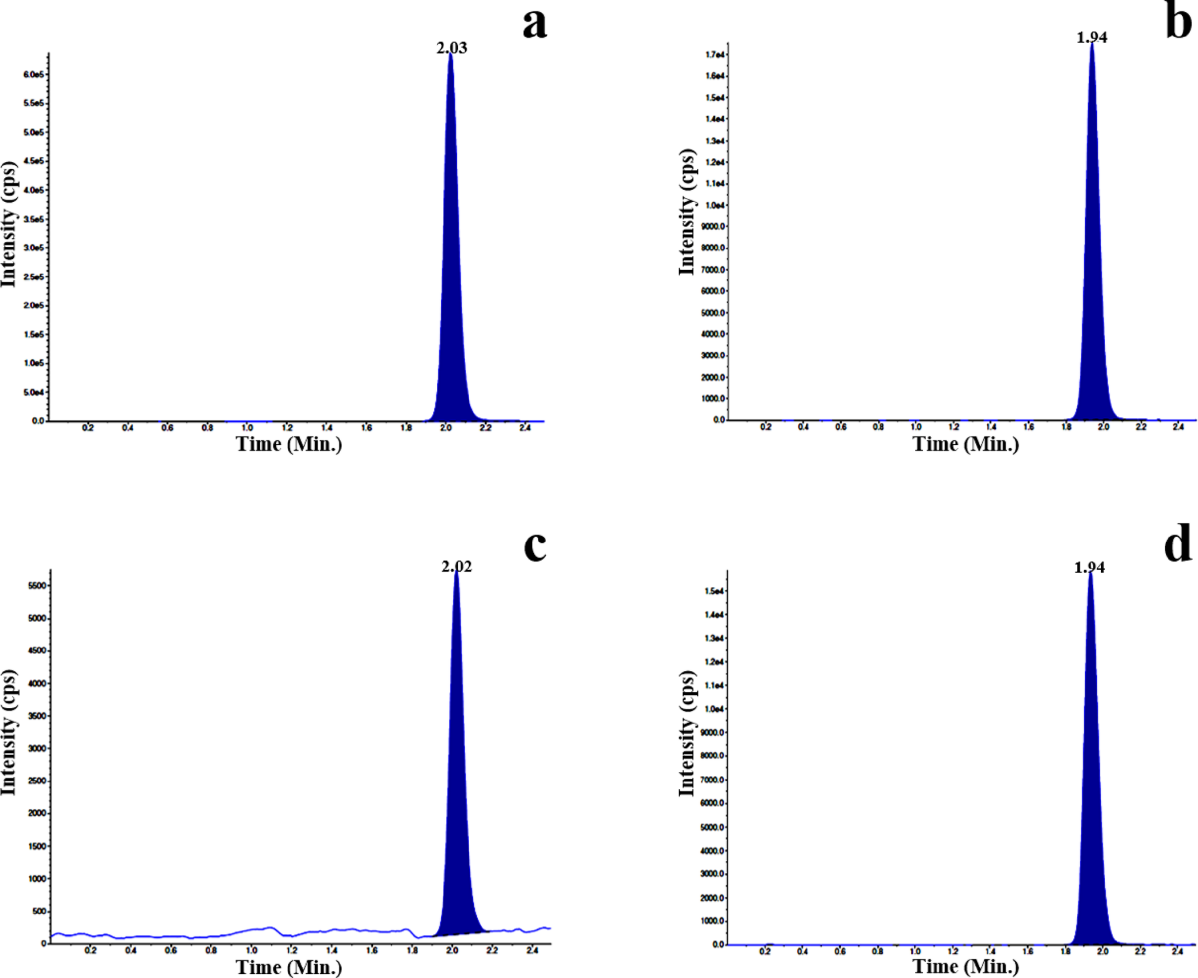
Representative chromatograms of standard quercetin (a) and hydrochlorothiazide (b) in plasma; quercetin in plasma after oral administration (2 h) of optimized quercetin-loaded nanoemulsion (c); hydrochlorothiazide during sample analysis (d).

The calibration curve was linear in the 5–5000 ng mL^–1^ concentration range. It was plotted by keeping the peak area ratio of QT to HCZ (*Y* axis) vs QT concentration (*X* axis) with the calibration equation *y* = 0.0063*x* + 0.0592 (*R*^2^ = 0.995). The LC-MS/MS representative chromatograms of plasma containing standard QT (Figure 4a) and HCZ (Figure 4b) in rabbit plasma are illustrated. Further, the chromatogram of QT in plasma that was obtained after 2 h of oral-administered QTNE (30 mg/kg) is illustrated in Figure 4c and HCZ during sample analysis is illustrated in Figure 4d. The respective chromatograms exhibited no interference by endogenic plasma constituents. The LLOQ for QT was found to be 5.1440 ng mL^–1^; at the LLOQ, the precision and accuracy values were 2.38% and 100.32%, respectively. The inter- and intra-day results for the precision and accuracy are tabulated in Table 1. The accuracy (intra-day) of QT was in the range of 93.80–100.27%, with precision (%RSD) ≤4.96%. The accuracy (inter-day) of QT was in the range of 93.14–99.90%, with precision (%RSD) ≤5.62%. The extraction recoveries of QT were estimated at three, 11.2560, 1880.9660, and 3761.9320 ng mL^–1^, and the absolute mean extraction efficiency (%) of QT was in the range of 87.99–90.62%, with %RSD ≤ 9.87 (Table 2). The matrix effect values were estimated by investigating the LQC (11.2560 ng mL^–1^), MQC (1880.9660 ng mL^–1^), and HQC (3761.9320 ng mL^–1^) samples. The values of average matrix effects were 101.03, 97.52, and 99.43% for LQC, MQC, and HQC, respectively, signifying no significant matrix effects. The results of freeze–thaw cycles for the QC samples of LQC, MQC, and HQC exhibited accuracies of 97.69, 94.52, and 98.90%, respectively, whereas the accuracies of freshly prepared QC samples of LQC, MQC, and HQC were 103.13, 99.84, and 102.53%, respectively, indicating apt freeze–thaw stability.

**Table 1:** Intra- and Inter-Day Precision and Accuracy of Quercetin in Rabbit Plasma (*n* = 3)

spiked concentration(ng mL^–1^)	mean concentration(ng mL^–1^)	precision(RSD)	accuracy(%)
Intra-day			
LQC (11.3560)	11.1196	0.5517	4.96
MQC (1880.9660)	1882.1775	9.9077	0.53
HQC (3761.9320)	3690.9294	142.9449	3.87
Inter-day			
LQC (11.3560)	10.5775	0.5951	5.62
MQC (1880.9660)	1879.1700	14.9127	0.79
HQC (3761.9320)	3739.3941	115.3848	3.08

**Table 2:** Extraction Recovery of Quercetin and Internal Standard in Rabbit Plasma (*n* = 3)

	QT		internal standard (HCZ)	
QC	recovery (%)	RSD (%)	mean recovery (%)	mean RSD (%)
LQC	89.21	9.87	89.58	3.14
MQC	90.62	2.72		
HQC	87.99	2.83		

### 3.2 Pharmacokinetics Study

**Figure 5:**
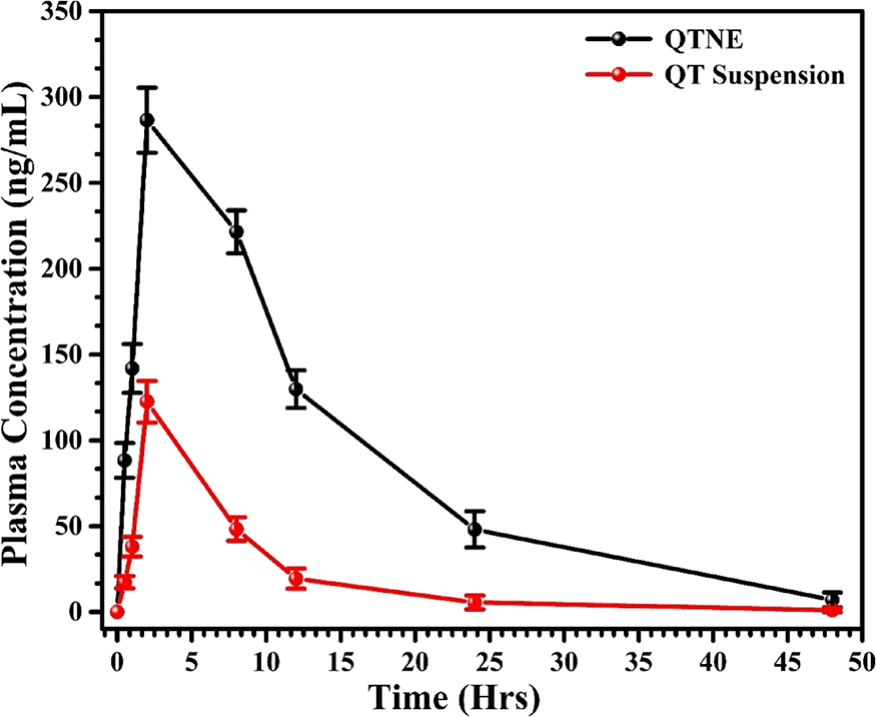
Comparative plasma concentration-time profile of optimized quercetin-loaded nanoemulsion (QTNE) and quercetin suspension (QTS).

The drug carriers usually move into the organs, in the initial phase, rather than going to the targeted sites, and further, they reach the targeted organs through the systemic circulation. The process of drug absorption from the site of administration plays a crucial role before its passage into systemic circulation.^22^ The extent and rate of drug absorption depend upon various factors, including the route of administration, physiological conditions of the drug absorption site, and the mechanism of drug absorption. In oral administration, before moving into systemic circulation, the drug passes through various layers of epithelial cells of the GIT, leading to difficulties in the absorption of the drug.^24^ In addition, poor solubility and/or low permeability of drugs leads to the problem of poor absorption and low bioavailability, and these limitations can be overcome by encapsulating these drugs within specific nanocarrier systems. The validated bioanalytical method was effectively employed to quantify the amount of QT in rabbit plasma. The pharmacokinetic parameters followed by oral administration of QTNE and QTS were determined using GastroPlus software. Finally, the results for the relative PDCT profile of QTNE and QTS are illustrated in Figure 5.

Additionally, the results of the plasma-drug concentration time profile of QTNE and QT suspension were evaluated for non-compartmental and compartmental analysis (1C, 2C, and 3C) using the PkPlus module (Table 3).

**Table 3:** Pharmacokinetic Parameters of Optimized Quercetin-Loaded Nanoemulsion (QTNE) and Quercetin Suspension (QTS) Generated after Non-Compartmental and Compartmental Analysis Using the PkPlus Module of GastroPlus Software^a^

	QTNE				QTS			
PK parameters	NCA	1C	2C	3C	NCA	1C	2C	3C
*C*_max_ (ng mL^–1^)			286.51(6.6)				122.56(9.93)	
*t*_max_ (h)			2				2	
AUC_0–*t*_ (ng h mL^–1^)	4249				976			
AUC_0–∞_ (ng h mL^–1^)	4337				992			
AUMC (ng h^2^ mL^–1^)	53,620				8718			
*t*_1/2_ (h)		8.047(27.33)	9.05			6.888(92.44)	11.09	
MRT (h)	12.36				8.79			
*K*_el_ (h^–1^)	0.08				0.068			
*K*_a_ (h^–1^)		0.633(38.75)	0.323(22.23)	0.323		0.917(147.64)	0.364(55.46)	0.364(55.46)
*K*_10_ (h^–1^)		0.086(27.33)	0.159(14.7)	0.15(11.3)		0.101(92.44)	0.266(40.56)	0.24(26.06)
*K*_12_ (h^–1^)			0.087(97.56)	0.087(97.56)			0.074(163.8)	0.074(163,96)
*K*_21_ (h^–1^)			0.157(103.05)	0.158(103.04)			0.085(188.12)	0.085(187.88)
*K*_13_ (h^–1^)				9.47 × 10^–3^(100.41)				0.026(329.05)
*K*_31_ (h^–1^)				7.53 × 10^–6^(99.89)				3.48 × 10^–5^(329.05)
*R*^2^		0.917	0.954	0.954		0.588	0.726	0.726
AIC		–28.807	–29.837	–25.836		–9.087	–10.539	–6.539
SC		–28.568	–29.440	–25.280		–8.848	–10.142	–5.982

a Values represented in parentheses represent the percent coefficient of variation (%CV) for respective parameters.

**Figure 6:**
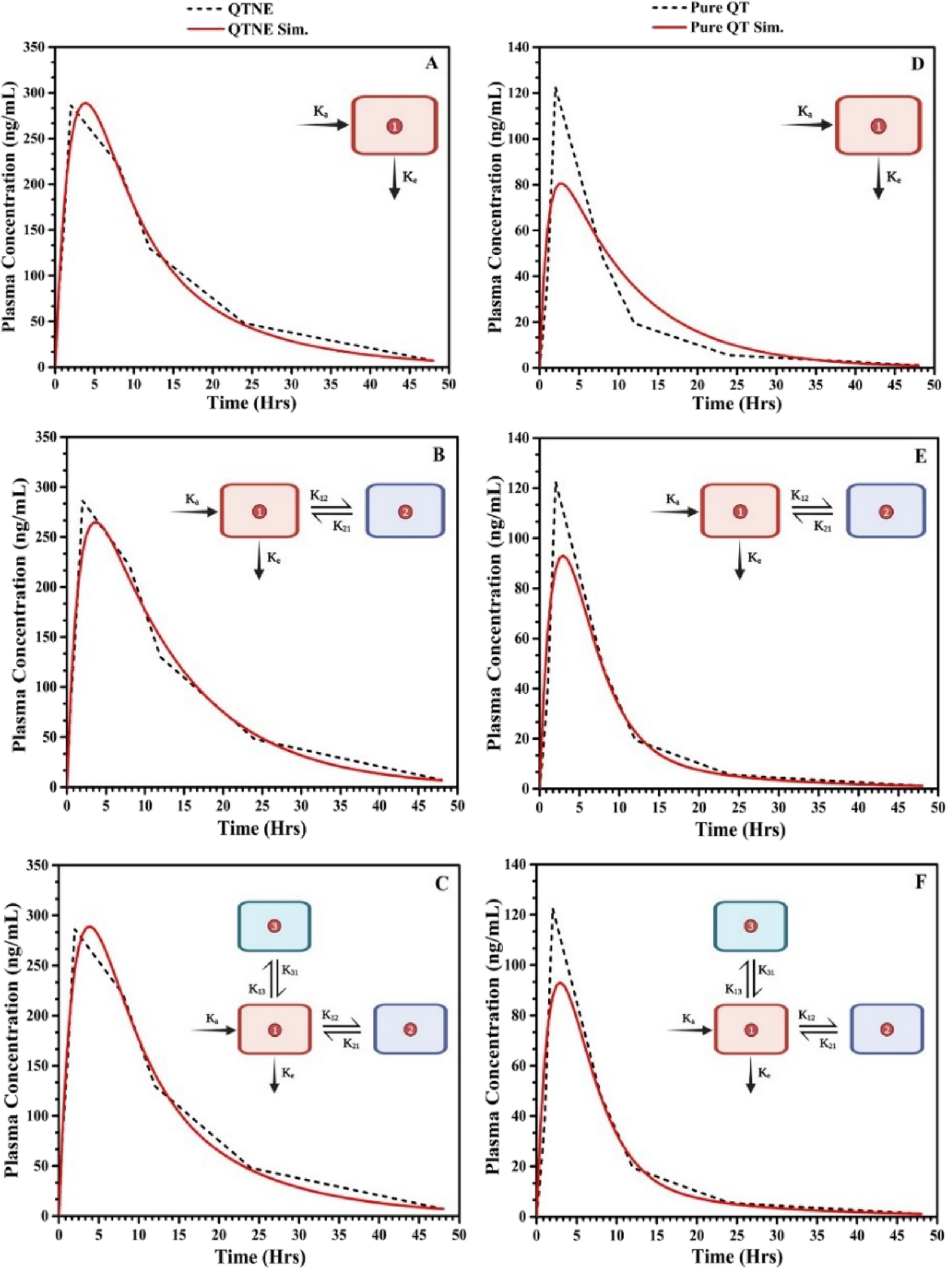
Plots representing the overlay of the true (black dotted) and simulated (red curve) plasma drug concentration-time profile of optimized quercetin-loaded nanoemulsion: (A) one-compartment model, (B) two-compartment model, (C) and three-compartment model; and quercetin suspension: (D) one-compartment model, two-compartment model (E), and three-compartment model (F). The insets display the corresponding pharmacokinetic models.

The non-compartmental method does not undertake any specific compartmental model and provides precise results; thus, it is considered more versatile and is widely applied in bioequivalence studies.^25^ The pharmacokinetic compartment modeling produces necessary information associated with the fate of a drug to time. The compartment modeling exhibits advantages compared to NCA in predicting the drug concentration at any specified time. The 1C pharmacokinetic model is the simplest compartmental pharmacokinetic model in which the whole organism is considered as a single compartment where the distribution of the drug is homogeneous and instantaneous (Figure 6A).^26^ On the other hand, in the 2C pharmacokinetic model (Figure 6B), the body is divided into two compartments: the central and peripheral. The first compartment (central compartment) comprises the plasma and tissues where the drug distributes almost instantaneously. The second compartment (peripheral compartment) comprises tissues where the drug distributes slowly.^27^ In the 3C pharmacokinetic model, the body is divided mainly into central and two peripheral compartments. The central compartment (compartment 1) is connected with the second and third compartments (peripheral compartments) where the drug distributes slowly as compared to the central compartment^28^ (Figure 6C). The correlation and fitness of the superposition among the true and simulated PDC (1C, 2C, and 3C models) and the corresponding compartment models (added as inserts) are illustrated in Figure 6.

Generally, after absorption, the drug moieties vacate the administration site to enter the central compartment and are then released into the peripheral compartment followed by distribution and finally eliminated irreversibly. This approach to drug movement from one compartment to another is considered by transfer rate constants, termed micro-constants.^29^ An upsurge in *C*_max_ from 122.56 ng mL^–1^ (QTS) to 286.51 ng mL^–1^ (QTNE) (2.34-fold), AUC_0–48_ from 976 ng h mL^–1^ (QTS) to 4249 ng h mL^–1^ (QTNE) (4.35-fold), and AUC_0–∞_ from 992 ng h mL^–1^ (QTS) to 4337 ng h mL^–1^ (QTNE) (4.37-fold) confirmed a significant improvement in the oral bioavailability of QT when delivered using the NE system. In a pharmacokinetic study performed with male albino Wistar rats using the HPLC technique, *C*_max_ of QTNE was 3.64-fold higher than pure QT; also, the QTNE exhibited a delayed *t*_max_ compared to pure QT.^30^ The results signified that QTNE improved pure QT’s bioavailability and sustained release properties. In another study, a single dose of QTNE and pure QT (dispersed in 0.3% sodium carboxymethyl cellulose) was administered orally (40 mg/kg) to mouse models. The results showed that QTNE exhibited improved solubility and permeability with *C*_max_ (10.04 ± 2.010 μg mL^–1^) values, which were 28.7-fold higher than the pure QT.^31^

Studies have shown that the encapsulation of QT within polymeric coats has also helped increase its oral bioavailability. Penalva et al. reported that the value of *C*_max_ of QT-loaded zein nanoparticles (Q-ZNP) was 2.5 times higher than the QT-PEG400/water solution studied in Wistar rats (25 mg/kg) using the HPLC method, ensuing an improved oral bioavailability of QT.^32^ In a recent study, researchers reported that QT’s oral bioavailability was significantly higher when encapsulated within the shell of zein-caseinate NPs (QZCNs) than caseinate-chitosan double layering (QZCCNs). The oral bioavailability of QT, studied in the rat model using the HPLC method, was enhanced in both QZCN- and QZCCN-treated groups by 2.34 and 1.89 times, respectively.^15^ Shen and co-researchers analyzed pharmacokinetic parameters of QT hybrid nanocrystals (QHNs; 90 mg/kg) with varying sizes (280 and 550 nm) in plasma and tissue homogenates of treated male Sprague–Dawley (SD) rats using the HPLC technique. Both types of QHNs showed improved oral bioavailability of QT; however, QHNs-280 exhibited higher AUC_0–*t*_ (82.40 h μg mL^–1^) and *C*_max_ (3.70 mg mL^–1^) than QHNs-550 with AUC_0–*t*_ (50.32 h μg mL^–1^) and *C*_max_ (2.05 mg mL^–1^), showing higher oral bioavailability of QHNs-280.^33^

The NE system mainly comprises oil and/or surfactant, which assists in enhancing the drug solubility and permeability, decreasing gastric degradation, and overcoming the issues of first-pass metabolism.^34^ Also, the smaller size of NE allows them to permeate deep within the tissues and prolong their circulation, leading to improved bioavailability of the encapsulated drug/s.^35^ In addition, NE protects the encapsulated drug/s from hydrolysis, oxidation, and volatilization.^36^ Drug delivery is affected by various factors, including solubility, enzymes, pH of GIT, ionic strength, consumed food constituents, dissolution rate, absorption window, and residence time.^37^ Sha et al. stated that the possible mechanism for the increased oral absorption of the QT-loaded NE was due to the surfactants used in NE formulation, which assisted the opening of tight junctions (paracellular pathway), whereas aqueous dispersion of QT showed precipitation in the cell monolayer.^38^ Furthermore, pancreatic lipases specifically digest the oil/lipid, major compositions of NE, on the apical margins of enterocytes. Later, the solubilized QT may permeate through the epithelial cells of the intestine *via* passive diffusion. NEs (∼20 nm) might permeate directly through the intestinal membrane or be captivated into the enterocytes through caveola- or clathrin-facilitated macropinocytosis and enterocytosis.^39,40^

Our study used CR RH 40 (ethoxylated hydrogenated castor oil) as a surfactant for preparing the NE system.^3,4^ It is well known for its effective inhibitory effects against the P-gp efflux pump, leading to enhanced absorption of the encapsulated drug.^41^ MRT is described as the arithmetic mean of the total time taken by a drug to remain in the body before elimination. It is an important pharmacokinetic parameter as it provides exact information regarding the existence of a drug molecule within the body, *i.e.*, some drug molecules last for a concise period, and others last longer.^42^ In our study, the MRTs of QTNE and QTS were found to be 12.36 and 8.79 h, respectively (Table 3), showing a prolonged circulation time of QT from QTNE in the body. The results of the compartmental analysis for QTNE demonstrated that the 2C model was selected as the best-fit model based on the lower AIC (−29.837) and SC (−29.440) values with higher *R*^2^ (0.954) values. These results indicated that the drug might not diffuse rapidly within the peripheral compartments. Furthermore, similar results of the compartmental analysis for QTS were noticed, suggesting the 2C model as a best-fit model with AIC, SC, and *R*^2^ values of −10.539, −10.142, and 0.726, respectively.

### 3.3 *In Vitro*–*In Vivo* Correlation (IVIVC)

IVIVC includes a set of predictive mathematical models describing the correlation between dosage forms *in vitro* drug release profile and significant *in vivo* response. It is an important design component for modified-release dosage forms.^43^ A dosage form’s *in vitro* and *in vivo* properties are correlated and represented mathematically through various linear or/and non-linear methods.^23^ Generally, the linear method is represented mathematically by using the convolution or/and deconvolution approaches. The numerical convolution or/and deconvolution approaches are usually considered because they do not create any pharmacokinetic model presumptions. Furthermore, the *K*_a_ (*in vivo*) can be estimated using a compartmental approach if the pharmacokinetic parameters of the drug are known.^44^

**Figure 7:**
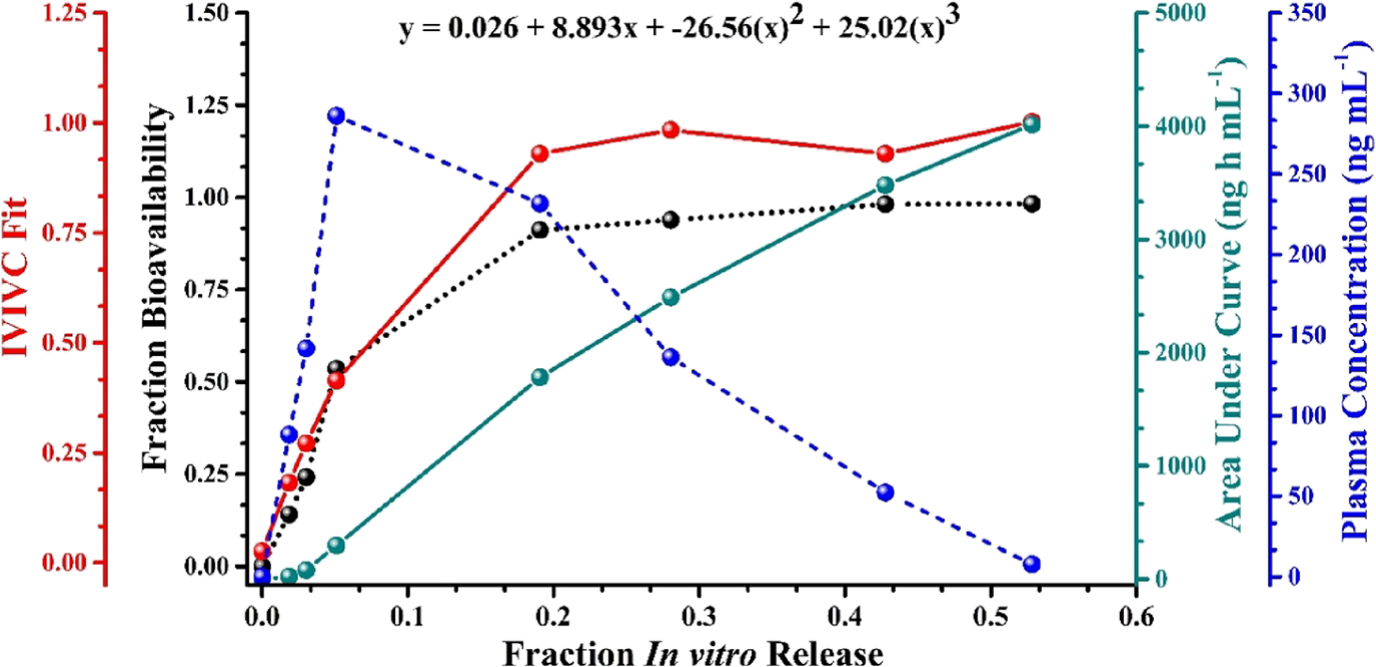
*In vitro*–*in vivo* correlation curves between fraction *in vitro* release and *in vitro*–*in vivo* correlation (IVIVC) fit, fraction bioavailability, area under curve (ng h mL^–1^), and plasma concentration (ng mL^–1^). The inset equation represents the fit curve equation, where *x* is the fraction *in vitro* release and *y* is the fraction absolute bioavailability.

In the present study, the IVIVC model was established using the *in vitro* dissolution (reported in our previously published work)^4^ and *in vivo* pharmacokinetic profile of QTNE and was analyzed using GastroPlus software. The correlation between the parameters on the *Y* axis (IVIVC fit, fraction bioavailability, AUC, and plasma concentration) and *X* axis (fraction *in vitro* release) for QTNE is illustrated in Figure 7. The IVIVC data were fitted using the third-order polynomial (eq 3)
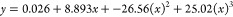
3where *x* and *y* signify the fraction *in vitro* release and fraction absolute bioavailability of the drug, respectively.

Furthermore, the correlation function is related to the Loo–Riegelman (2C model) with percent prediction error (%PE) between the observed (Obs.) and predicted (Pred.) values of *C*_max_ and AUC_0–*t*_. The statistical analysis of reconstructed PDCT profiles from the convolution data is represented as *R*^2^, SEP, MAE, and AIC. The %PE was calculated as per eq 4(22)
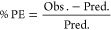
4

The observed and predicted values for *C*_max_ of QTNE were 287 and 256 ng mL^–1^, respectively, with a %PE of 10.80. A similar method was used to determine the observed and predicted values for AUC_0–48_ of QTNE, and they were found to be 4020 and 3897 ng h mL^–1^, respectively, with a %PE of 3.06%. Based on the statistical analysis of reconstructed PDCT profiles indicating a high value of *R*^2^ (0.998) and low values of SEP (4.537), MAE (2.759), and AIC (42.38), the Loo–Riegelman method (2C model) was selected as the best-fit deconvolution model. The IVIVC exhibited a similar approach as that of the pharmacokinetic profile of QTNE, showing the 2C model as the best-fit model.

## 4 Conclusions

A rapid, specific, sensitive, and reproducible LC-ESI-MS/MS method was developed and validated to determine QT in rabbit plasma. HCZ was employed as an internal standard, and the linearity was determined in a concentration range of 5–5000 ng mL^–1^, with *R*^2^ = 0.995. The higher levels of *C*_max_ and AUC_0–*t*_ of the QTNE signified an improved oral bioavailability (2.34-fold) of QT compared to QTS, probably due to enhanced solubility, absorption, and residence time of QT. GastroPlus simulation software showed superior prediction accuracy used to estimate various pharmacokinetic parameters (compartmental and non-compartmental methods) of the developed QTNE. IVIVC was established using the IVIVC module of GastroPlus software. Analysis of the statistics generated from various IVIVC approaches revealed that Loo–Riegelman (2C model) was the best-fit model for the IVIVC of QTNE with *R*^2^ = 0.998. The study revealed that the QTNE is a potential nanocarrier for effectively delivering QT with improved oral bioavailability. The established bioanalytical method can be efficiently employed in preclinical/clinical studies to quantify QT in animal plasma.

## Abbreviations

1C: one-compartmental analysis
2C: two-compartmental analysis
3C: three-compartmental analysis
AIC: Akaike information criterion
AUC_0/*N*_: area under the plasma drug concentration-time curve from time zero to infinity
AUC_0/*t*_: area under the plasma drug concentration-time curve from time zero to time *t*
AUMC: area under the moment curve
*C*_max_: peak plasma drug concentration
GIT: gastrointestinal tract
*K*_a_: absorption rate constant of the drug
*K*_e_: elimination rate constant
LR2C: Loo–Riegelman two-compartment model
MAE: mean absolute error
MAPPE: mean absolute percent prediction error
MRT: mean residence time of the drug
NCA: non-compartmental analysis
PDCT: plasma drug concentration-time profile
QTS: quercetin suspension
QTNE: optimized quercetin-loaded nanoemulsion
*R*^2^: correlation coefficient
SC: Schwartz criterion
SEP: standard error of prediction
*t*_1/2_: half-life of the drug
*t*_max_: time to achieve peak plasma drug concentration
P-gp: P-glycoprotein
MRP2: multidrug resistance-associated protein 2
CyP450: cytochrome P450
